# 11,000 years of craniofacial and mandibular variation in Lower Nubia

**DOI:** 10.1038/srep31040

**Published:** 2016-08-09

**Authors:** Manon Galland, Denis P. Van Gerven, Noreen Von Cramon-Taubadel, Ron Pinhasi

**Affiliations:** 1 School of Archaeology and Earth Institute, University College Dublin, Belfield, Dublin 4, Ireland; 2 Muséum national d’Histoire naturelle – UMR 7206 - CNRS, 75016 Paris, France; 3 University of Colorado, Boulder, United States; 4 University at Buffalo, SUNY, Department of Anthropology, Buffalo, United States

## Abstract

The transition to agriculture was a key event in human history. The extent to which this transition is associated with biological changes in different world regions remains debated. Cultural and osteological records in Lower Nubia throughout the Holocene have been interpreted as a result of *in situ* differentiation or alternatively as migratory events and possible admixture with surrounding populations. Here we investigated the patterns of craniofacial and mandibular variation from Mesolithic hunting-gathering to late farming, a period spanning 11,000 years. We analyzed 102 adult specimens spanning five cultural horizons: Mesolithic, A-group, C-group, Pharaonic and Meroitic, by means of 3D geometric morphometric methods, in order to assess shape variation and diachronic patterns at the transition to farming and in subsequent periods. Our results highlight a strong morphometric distinction between Mesolithic hunter-gatherers and farmers as well as differences between transitional and intensive farmers in mandibular variation which is consistent with differential impact of selective pressures on different regions of the skull. This study corroborates a major biological change during the transition from hunting to farming, supporting the masticatory-functional hypothesis for the mandible and suggesting population continuity among farming populations throughout the Holocene based on the overall shape of the cranium.

The history of prehistoric human populations from Lower Nubia during the Holocene has drawn great interest in anthropology for the richness of archaeological findings and the opportunity to examine the process of subsistence change from hunting and gathering to farming through time. The shift to agriculture was a key event in human history driving major biological and cultural change globally^1^,^2^. However research indicates that the nature and timing of this transition varied across world regions^1^,^2^,^3^,^4^. In Lower Nubia, biological affinities, *in situ* microevolutionary processes, population movements and the relationships among people from the Mesolithic to the Meroitic period remains highly debated^5^,^6^,^7^. Here we applied 3D geometric morphometric methods to investigate both cranial and mandibular variation among Mesolithic hunter-gatherers and early and late farmers.

Lower Nubia (or northern Nubia^8^) covers the area extending from southern (or Upper) Egypt to northern Sudan along the Nile valley, from the first to the second cataract^9^ (Fig. 1). Different cultural horizons, analysed in the present study, have been defined based on archaeological evidence of varying subsistence strategies: Mesolithic, A-group, C-group, Pharaonic and Meroitic.

Mesolithic Nubians (11,000–8,000 BCE) are associated with a hunting and gathering adaptation, particularly focussed on the exploitation of large game hunting plus some fishing and seed collecting^5^,^10^. They were characterized by a low density and dispersed population^11^.

A-group Nubians (3,300–2,800 BCE) represent a transition from Mesolithic to Neolithic^9^. They were semi-nomadic herders and rudimentary agriculturalists with evidence of domesticated animals and grains plus extensive fishing, hunting and gathering^8^,^9^,^10^,^12^. A-group Nubians could be descendants of Early Mesolithic/Late Neolithic Nubians or related to populations from Upper Nubia^13^. Archaeological evidence suggests that A-group Nubians represent an indigenous cultural development^9^,^13^. The main issue regarding this cultural horizon is the presence of a major hiatus around 5,000 to 8,000 years between Mesolithic and A-group as evidenced by a lack of well-preserved skeletal remains in the archaeological record between the Mesolithic and A-group horizon, making estimation of their ancestry difficult^7^,^9^,^13^.

C-group Nubians (2,300–1,800 BCE) share numerous cultural features with A-group such as presenting a mixed economy^10^,^13^,^14^. They also present an intensified agricultural regime and a much more complex culture^9^,^15^. Many studies suggest that C-group is a continuation of the A-group^9^,^10^,^13^,^14^ although there is a gap of a few centuries between the A- and C-group cultural horizons. The lack of archaeological evidence during this gap could be the result of political pressure from the Egyptian Old Kingdom pushing populations from Lower to Upper Nubia as well as of ecological factors associated with the desiccation of the Nile River leading to a reduction of the available resources^8^,^9^. Another hypothesis proposes that C-group Nubians are not only descendants from A-group but encompasses new people from the surrounding area^7^,^8^.

Pharaonic Nubians refer to populations living during the expansion of the Egyptian New Kingdom (1,550–1,070 BCE) from the eighteenth to the twentieth dynasty^8^,^9^. They are characterized by a very complex agriculture (use of irrigation systems) and animal domestication^8^. Their biological and ethnic affiliation remains debated with archaeological evidence showing a mix of Nubian and Egyptian cultures: they could be C-group assimilated to the Egyptian culture, “Egyptianized Nubians”^9^ or Egyptian migrants^8^,^9^,^14^.

Meroitic Nubians (100 BCE–350 CE) are associated with a fully farming and animal husbandry adaptation with a highly sophisticated culture displaying evidence of seasonal strategies, trade, and use of the waterwheel which increased the potential of food production and led to larger population sizes^9^,^10^,^14^,^16^. For almost a thousand years between the Pharaonic and Meroitic horizons, there is a lack of archaeological evidence known as the “Nubian Hiatus”, which is probably due to a drop of the Nile River making the area uninhabitable^9^,^13^. Debates persist whether Meroitic Nubians were descendants of C-group and/or Pharaonic Nubians or whether they represent a new ethnic group^8^.

Several models have been proposed to explain the biological variation among Nubian populations during the Holocene. The “population continuity” hypothesis suggested by Greene^17^, proposed population continuity from the Mesolithic through to the Christian period based on the analysis of craniofacial and dental data. Despite the fact that there is no physical continuity in occupation with some gaps in the archaeological record as mentioned above, this model emphasizes cultural and biological continuity with no evidence of a major population replacement over a period of 12,000 years^9^,^17^. This hypothesis has received support from different studies based on cranial measurements^6^,^9^,^18^,^19^, dental traits^17^,^20^,^21^,^22^ and archaeological artefacts^13^. Carlson and Van Gerven^6^ highlighted the importance of selective pressures in Lower Nubians proposing the “masticatory-functional hypothesis”, which suggests that a reduction in functional demands relating to mastication led to an alteration of facial morphology and a reduction of dental and mandibular dimensions in later farming populations. This hypothesis supposes that the shift to agriculture led to an increasing reliance on softer and processed foodstuffs, which is corroborated by independent evidence from dental microwear as well as from molecular studies of crop plant, and that consequently agriculturalists experience shorter and less intensive chewing^2^,^5^,^6^. However other studies found the morphological divergence among groups to be too great to be accounted for by continuity or adaptive change, suggesting the presence of a post-Pleistocene biological discontinuity due to population movements along the Nile corridor. This “population influx” hypothesis is mainly supported by dental variation studies^7^,^23^,^24^ but has also some support from cranial^25^ and postcranial analyses^26^.

Cranial morphology is shaped by both neutral evolutionary forces and adaptation to extrinsic factors^27^,^28^,^29^,^30^,^31^. While a consensus has emerged in recent years that global patterns of cranial shape variation can be explained to a large extent on the basis of a neutral model of population diversification^27^,^28^,^29^,^31^, previous research has emphasized that a response to modifications in masticatory behaviours could significantly influence overall skull morphology^6^,^32^,^33^,^34^,^35^,^36^,^37^ and the mandible in particular^6^,^35^,^36^. As such, craniometric data can be employed as a proxy for population biodistance, facilitating the investigation of both population history and adaptation to selective pressures in order to comprehend the biological processes involved during the transition from hunter-gathering to farming. Focusing on the Nile corridor is particularly interesting because of the richness of its archaeological and osteological evidence, the presence of cultural interactions and development of complex societies prior and during the time of the Egyptian Kingdom. Here, using 3D geometric morphometrics for the first time to quantify and visualize overall cranial size and shape variation across Nubian prehistory, our study aimed to evaluate both cranial and mandibular morphological patterns among Nubian series from 11,000 BCE to 350 CE (Table 1). Specifically, we tested the following hypotheses:

- The population continuity hypothesis, according to which, we would expect to observe a relative homogeneous morphological pattern among all chrono-cultural groups with no clear distinction based on chronology and strong overlap in morphological variability among all groups.
- The masticatory-functional hypothesis, according to which, we would expect strong morphological differences localized in the mandible between dietary groups (hunter-gatherers, early farmers, intensive farmers) as well as a significant decrease in size and robustness of the face between hunter-gatherers and farmers.
- The population influx hypothesis, according to which, we would expect a significant differentiation between the Mesolithic specimens and the agricultural groups. We expect to see these morphological differences most strongly in overall skull morphology not only in the region related with masticatory processes but especially in the cranial morphology which is assumed to reflect population history more than dietary adaptation.

## Results

Overall cranial and mandibular shape variation underlines a strong distinction between the Mesolithic specimens and the other four cultural groups. Patterns of between-group differentiation among the four post-Mesolithic groups are more complex and differ between analyses based on either the cranium or the mandible.

Regarding size variation, Mesolithic Nubians show a larger average size for both the cranium and the mandible (Fig. 2). However significant differences in size between Mesolithic Nubians and all other cultural group were only found for the mandible (Supplementary Tables S3 and S4). No significant differences in size were detected among the early and late Nubian farmers for either the cranium or the mandible. Crania with a bigger centroid size corresponding to Mesolithic specimens have relatively wider zygomatic processes and smaller faces and vaults (Fig. 3). Bigger mandibles, also corresponding to Mesolithic specimens, had a wider and more robust corpus compared with smaller mandibles (Fig. 3). However allometric effects are very low for both the cranium and the mandible (Supplementary information; Tables S5 and S6).

Concerning shape variation, the distinction between Mesolithic specimens and the remaining groups is very clear on the first axis of both between-group principal components analyses performed on the cranium and on the mandible explaining 53% and 60% of the total variance, respectively (Fig. 4). Shape differences are pronounced between Mesolithic hunter-gatherers and post-Mesolithic early and late farmers, particularly in the mandible. Mesolithic Nubians have shorter, wider and more upright ramus and coronoid process, longer mandibular condyle and deeper, wider and upright corpus. They also exhibit lower and wider cranial vaults, shorter and wider faces, much wider zygomatics, more pronounced alveolar prognathism, more projected glabella, longer mastoid processes, lower and wider orbital apertures and a smaller nasal aperture. Mahalanobis distances between Mesolithic Nubians and any other cultural group were also larger for both the cranium and the mandible (Table 2; Fig. 5).

There is no such clear morphological differentiation between early and late farmer groups. Again shape differences are more pronounced for the mandible than for the cranium. Pharaonic and Meroitic Nubians tend to be distinguished from A-group and C-group specimens by exhibiting shorter, wider and less upright corpus. C-group specimens, contrary to the ones associated with Pharaonic and Meroitic cultural horizon, tend to have higher vaults, longer mastoid processes, smaller nasal apertures and slightly narrower faces. A-group specimens are in an intermediate position. Mahalanobis distances for both the cranium and the mandible indicate that A-group Nubians are slightly closer to C-group Nubians than any other cultural horizon (Table 2).

The cultural groups differ significantly in both cranial and mandibular shape variation (Table 3). The coefficient of determination was slightly higher for the cranium than for the mandible (R^2^ = 12.6% and 9.9%, respectively) when examining group affinity versus shape variation using MANOVA. The same analysis computed using the three dietary groups also demonstrates that diet has a significant impact on cranial and mandibular shape variation. The coefficient of determination was slightly higher when early farmers (A-group and C-group) and late farmers (Pharaonic and Meroitic) are considered as two groups rather than being grouped together (R^2^ = 8.7% compared with 6.1% for the cranium and R^2^ = 8% compared with 5.7% for the mandible). However, when the Mesolithic sample was excluded, the results for the MANOVA were significant only for the mandible with a smaller coefficient of determination (R^2^ = 5% for the cultural groups and R^2^ = 2.7% for the dietary groups).

## Discussion

Our results clearly depict a strong craniofacial and mandibular distinction in size and shape components between Mesolithic hunter-gatherers and early and late farmers. Mesolithic Nubians are characterized by a greater overall size and more robust mandibles, faces and zygomatics. These observations are congruent with previous studies highlighting a reduction of the facial robusticity from Mesolithic to A-group Nubians^6^,^9^,^10^,^19^,^38^. However, there is no significant diachronic pattern of reduction in cranial vault height and length^6^,^9^,^10^,^19^. Mesolithic Nubians tend to have lower vaults, greater alveolar prognathism, more projected glabella, and lower and wider orbits^38^. However, major shape changes described here concern the shape of the face and mandible, i.e., cranial and mandibular regions involved in the masticatory process, which is in agreement with morphological patterns found among hunter-gatherer populations from other regions^33^,^35^,^36^,^39^. Facial and mandibular robusticity suggests the presence of heavy chewing muscles and larger teeth, morphological characteristics expected for populations whose subsistence strategy is based on hunting-gathering^6^,^34^. We also confirm a reduction of both craniofacial and mandibular sizes which is consistent with other studies at a worldwide scale^6^,^10^,^11^,^34^,^40^.

While cranial and mandibular patterns in our analyses both indicate a strong distinction of Mesolithic Nubians and close morphological affinities between early and late farmers, the two anatomical datasets differ in terms of the pattern of differentiation found among cultural horizons. In evaluating our results alongside the three hypotheses outlined previously, we can draw the following conclusions. Neither the cranial nor the mandibular results align with the predictions of the “population continuity” hypothesis throughout the entire chronological sequence. The MANOVA results consistently found significant distinction between the Mesolithic and the other four cultural groups, and between early farmers and late farmers in the case of the mandible. However our results do not suggest any strong morphological differentiation occurring through time from the A-group to Meroitic cultural horizons, which is in agreement with previous studies that report no drastic change in skeletal series spanning from the A-group to the Christian period^6^,^9^,^10^,^19^. Nevertheless, the morphological distances between the A-group and C-group samples are relatively smaller, which is consistent with the hypothesis that C-group Nubians are direct descendants from A-group Nubians^9^,^13^,^14^. Similarly, shared morphological affinities between Pharaonic and Meroitic specimens support the notion that Meroitic Nubians were descendants from Pharaonic Nubians^9^,^13^,^14^. These two groups present shorter, wider and less upright corpus and less high vaults, morphological patterns also found in other farming populations^36^. However, the hypothesis that Pharaonic Nubians were descendants of the C-group Nubians that assimilated into Egyptian culture^14^ is not supported by our results which do not show any particular close affinities between these two groups. All these observations are more congruent with the general hypothesis of a regional continuity from A-group (non-intensive farmers) to Meroitic Nubians (intensive farmers) underlined by both biological and archaeological evidence^6^,^7^,^9^,^10^,^13^,^19^,^32^. Although we detect a significant strong distinction between Mesolithic and later Nubians, given the potential impact of selective pressures plus the temporal gap that could have been as much as 4,700 years, we cannot totally reject the presence of a relative biological continuity since the Mesolithic with no evidence of major population replacement. In case of a discontinuity with external arrivals of new group(s), we could expect morphological changes in overall skull morphology and not only concentrated in cranial regions related to mastication.

Our results do support the predictions of the masticatory-functional hypothesis as we see a significant difference among all three dietary groups (hunter-gatherers, early farmers, and late farmers) in mandibular variation. Also, the pattern of size and shape differences among these groups is consistent with the masticatory-functional hypothesis, given a trend towards decreasing size and robusticity throughout the transition from hunter-gathering to farming. Our results also show that mandibular morphology, which is presumed to be subject to selective (or non-neutral) pressures^6^,^35^,^36^,^37^, continues to be significantly impacted by cultural and economic variation from 3,200 BCE to 350 CE in parallel with the evolution of a mixed subsistence strategy to one fully dependent on farming^9^,^13^,^14^. The evidence for the cranium is more ambiguous, as MANOVA found significant differences between Mesolithic hunter-gatherers and farming groups, but no significant differences among the four farming groups. However, the main differentiation between hunter-gatherers and farmers lay in the relative width and robusticity in the facial skeleton, which is consistent with adaptive change due to masticatory-induced phenotypic plasticity^6^,^41^.

Finally the cranial results align with the predictions of the “population influx” hypothesis at the point of transition from hunter-gathering to farming, as suggested by the strong distinction between the Mesolithic sample and all four later cultural groups, but little differentiation among the four farming groups. Although cranial shape variation is mainly concentrated on the face, there is a slight difference observed in the vault height between Mesolithic and post-Mesolithic samples (Fig. 4). MANOVA results could support the “population influx” hypothesis between the Mesolithic Nubians and the beginning of the A-group as well as a late regional continuity in cranial shape among the four post-Mesolithic groups, as the pattern of cranial variation is assumed to reflect population history rather than adaptation due to diversifying natural selection^28^,^29^,^31^,^36^. This is also reinforced by the Neighbor-joining analysis and Mahalanobis distances, which shows a strong difference between the Mesolithic and all later groups (Fig. 5; Table 2), but does not strongly distinguish among the farming groups, despite considerable temporal variation among them. According to the “population influx” hypothesis, the large differentiation that we see in both cranial and mandibular shape between the Mesolithic and the A-group could be explained by an arrival of new people at the advent of the shift from hunting-gathering to farming, with additional adaptive changes in the masticatory apparatus (especially the mandible) as the reliance on farming intensified. This is consistent with previous cranial studies which have identified sharp cranial shape differentiation in the early Neolithic period of Europe with the influx of farmers from the Near East and Levant^42^,^43^,^44^. However, the differences between the cranial and mandibular results suggest that as the reliance on farming intensified throughout the later periods, the shape of the mandible continued to be affected in accordance with the masticatory-functional hypothesis, generating systematic differences in mandibular shape between the Mesolithic, early farmer, and late farmer groups.

Taken together, our results suggest a dramatic shift in cranial morphology between the Mesolithic and the A-group cultural group, with little perceptible change in cranial shape between A-group and the later farming groups. In the case of the mandible, we observe the largest morphological change between the Mesolithic and the A-group, but also see morphological differentiation between the early farmers (A- and C-group) and the later farming groups (Pharaonic and Meroitic specimens). While we test three hypothetical scenarios in this study, as had been suggested on the basis of previous research, it is worth noting that these three scenarios are not mutually exclusive. *In situ* masticatory adaptation with population continuity or alternatively with an influx of people is theoretically possible as well as a migration of farmers and biological continuity thereafter. Our data support the functional-masticatory hypothesis and demonstrate that the biomechanical changes associated with dietary change strongly affect the mandible and aspects of facial morphology but not have any clear discernible effect on the rest of the cranial structure. Yet further studies including larger comparative samples and the combination of morphometric analyses with ancient DNA are needed to precise biological continuity between Mesolithic and A-group or the influx of people from outside Lower Nubia.

In conclusion, our study corroborates major biological changes during the transition from hunting to farming in the lower Nubian region. It also demonstrates the differential impact of selective pressures in different skull regions and highlights that the mandible, in contrast to the cranium, reflects adaptation to subsistence strategy rather than patterns of variation related to neutral evolutionary forces such as gene flow and genetic drift. Our results give strong support to both the masticatory-functional hypothesis with morphological changes especially concerning the mandible and cranial regions involved in the mastication, and the “population continuity” hypothesis among post-Mesolithic groups based on the overall shape of the cranium.

## Methods

### Material

**Figure 1:**
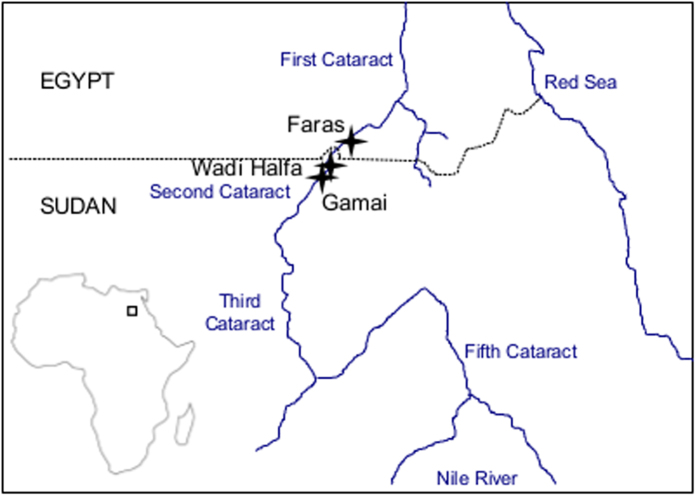
Map showing the area of the samples analysed (map created using Inkscape 0.91, www.inkscape.org).

**Figure 2:**
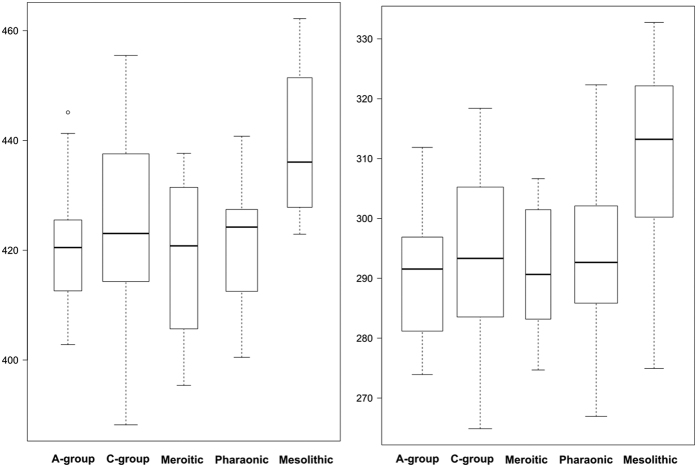
Boxplot of the centroid sizes by cultural horizon for the cranium (left) and the mandible (right).

**Figure 3:**
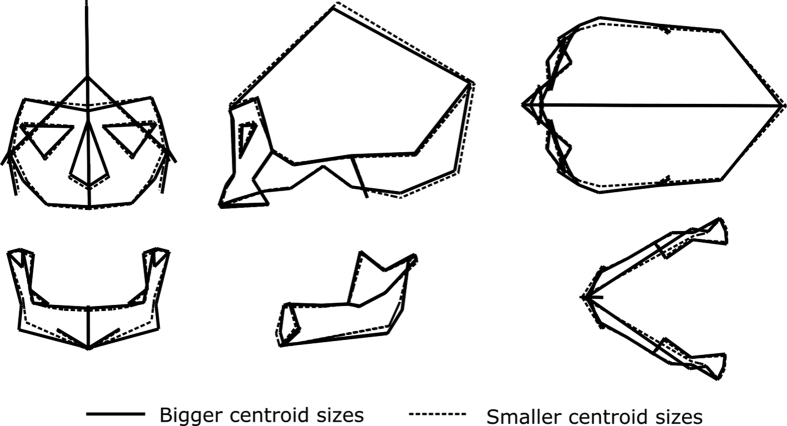
Impact of allometric effects observed on the crania and the mandibles.

**Figure 4:**
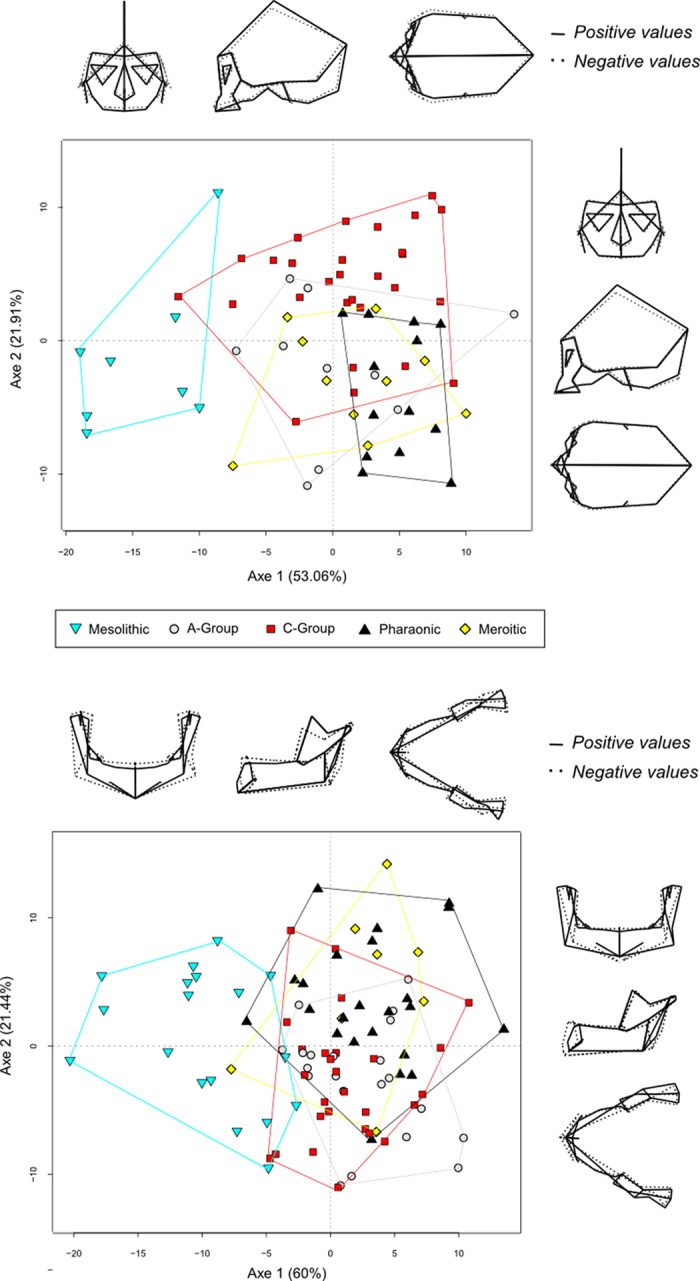
Between-group PCA based on Procrustes residuals of landmark configurations and shape differences observed for the cranium and the mandible.

**Figure 5:**
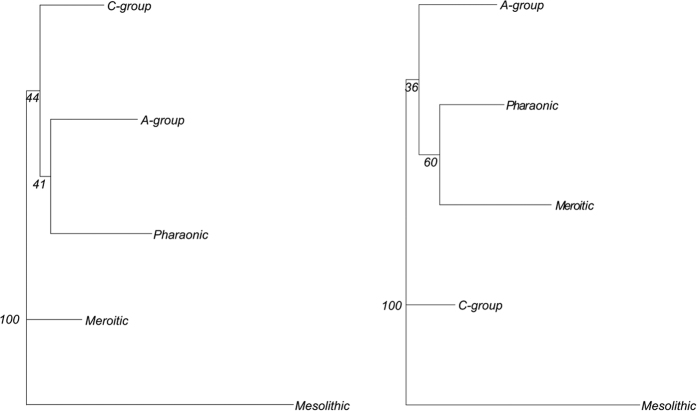
Neighbor-Joining trees based on Mahalanobis distances with bootstrap values for the cranium (left) and the mandible (right).

We analysed 69 crania and 97 mandibles of adult specimens of both sexes (Table 1). Data included in the present study were collected from skeletal remains associated with the Mesolithic (11,000–8,000 BCE), A-Group (3,300–2,800 BCE), C-Group (2,300–1,800 BCE), Pharaonic (1,800–1,200 BCE) and Meroitic (100 BCE–350 CE) cultural horizons from South Egypt/North Sudan located on the upper Nile between the first cataracts (Fig. 1). Mesolithic samples are from Wadi Halfa (Sudan) during the expedition to the Sudan led by the University of Colorado in 1964. A-group, C-group, Pharaonic and Meroitic samples are from Faras (Egypt) to Gamai (Sudan) during the Scandinavian Joint Expedition^8^. All specimens were anatomically considered adult with fully fused spheno-occipital synchodroses. Sub-adult specimens were excluded since their cranial morphology is not fully developed. Likewise adults displaying poor preservation or pathologies that could affect cranial shape were also excluded from the analyses. Crania and mandibles were collected from the same specimens and sex was equally balanced as much as possible. Sex was assessed with standard osteological techniques^45^.

### Morphometric data

**Figure 6:**
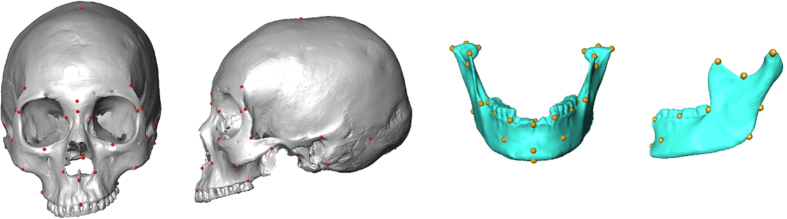
Facial and lateral views of a cranium and a mandible with 39 and 33 landmarks, respectively.

Each cranium and each mandible were digitized with a 3D surface scanner (Nextengine HD device; www.nextengine.com). One of the main advantages of 3D scanning is the possibility to get a virtual high-quality archive of osteological remains. Shape data were then collected in the form of three-dimensional coordinates of cranial landmarks. Thirty-nine and thirty-three homologous landmarks were respectively placed on each cranium and each mandible (Fig. 6) by a single observer (M.G.) using the Landmark Editor software^46^. Landmarks were chosen in order to reflect the overall skull shape and belong to all three types defined by Bookstein^47^. Anatomical description of all landmarks is presented in Tables S1 and S2.

Repeatability was assessed through six non-consecutive extractions on ten specimens. The intra-observer error for each landmark (mean 0.8 mm) was below reported standard errors in craniometrics^48^. Missing bilateral landmarks were estimated by mirroring-imaging^49^. Missing landmarks in the sagittal plane or bilateral landmarks missing on both sides were estimated by the Thin-Plate-Spline interpolation^50^ which permits the mapping of missing points from available landmark configurations to the incomplete specimens in a way that the deformation between complete and incomplete specimens is as smooth (i.e. bending energy is minimized) as possible^51^. As recommended by Couette and White^52^, all specimens have less than 20% of missing data. The estimation of missing landmarks was performed using R^53^,^54^.

### Geometric morphometrics and data processing

Landmark data was processed by 3D geometric morphometric methods. All craniofacial and mandibular landmark configurations were subjected to generalized Procrustes analysis (GPA^55^,^56^) which permits the extraction of geometric shape separate from overall (isometric) size. Landmark configurations were translated to a common centroid, scaled to unit centroid size, rotated by least squares fitting and then subjected to tangent space projection. This study focused on the symmetric component of shape variation, so all analyses are based on the averaged Procrustes coordinates of each landmark configuration and its mirror image in order to remove the effects of asymmetry^57^. All multivariate analyses were performed in R^53^. Differences in size between samples were studied by means of ANOVA tests and Tukey’s honestly significant differences (HSD). The pattern of shape variation and morphological differences were examined with between-group PCA which emphasizes among-group differences and is based on a between-groups variance-covariance matrix^58^. Among-group shape differences were visualized along the PCA axes. The PC scores from a normal total variance-covariance PCA required to account for 95% of the total variance were used to perform MANOVA as well as to generate the among-groups Mahalanobis distances. Neighbor-Joining trees (NJ^59^) were computed based on Mahalanobis distances using bootstrapping (1000 replications) to test the reliability of the clusters. MANOVA were then applied to test if there were significant morphological differences between samples from the five cultural horizons as well as from the three dietary groups. The allometric effect was tested using linear regressions using PC scores as dependent variables and the centroid size as independent variable.

## Additional Information

**How to cite this article**: Galland, M. *et al*. 11,000 years of craniofacial and mandibular variation in Lower Nubia. *Sci. Rep*. **6**, 31040; doi: 10.1038/srep31040 (2016).

## Supplementary Material

Supplementary Information

## Figures and Tables

**Table 1:** Samples and related information.

Sample	Dietary group	Date	N Crania (m/f/u)	N Mandibles (m/f/u)	Museum
Mesolithic	Hunter-gatherers (HG)	8,000–11,000 BCE	8 (2/6)	18 (8/9/1)	University of Colorado, Boulder
A-group	Early farmers (EF)	3,300–2,800 BCE	10 (4/5/1)	21 (9/11/1)	Panum Institute, Copenhagen
C-group	Early farmers (EF)	2,300–1,800 BCE	28 (14/12/2)	27 (12/13/2)	Panum Institute, Copenhagen
Pharaonic	Farmers (F)	1,800–1,200 BCE	13 (6/7)	23 (14/9)	Panum Institute, Copenhagen
Meroitic	Farmers (F)	100 BCE–350 CE	10 (5/4/1)	8 (4/3/1)	Panum Institute, Copenhagen

**Table 2:** Mahalanobis distances between cultural groups for the cranium (upper triangle) and the mandible (lower triangle).

Mahal. dist.	A-group	C-group	Meroitic	Pharaonic	Mesolithic
A-group	0	2.942074	3.142982	3.346135	6.408830
C-group	1.738055	0	2.477643	3.060727	6.016817
Meroitic	2.519526	2.727493	0	2.922747	5.738018
Pharaonic	2.451742	2.069805	2.346177	0	7.414554
Mesolithic	4.448209	3.766902	5.189174	4.030918	0

**Table 3:** Results of MANOVA performed on the cranium and mandible from the chrono-cultural groups.

		Cranium		Mandible	
MANOVA		R^2^	P	R^2^	P
Cultural groups	All samples	0.127	0.002 (**)	0.099	<0.001 (***)
	Without Mesolithic sample	0.072	0.395	0.050	0.009 (**)
Dietary groups	All groups (HG, EF, F)	0.087	<0.001 (***)	0.080	<0.001 (***)
	Farmers combined (HG, F)	0.061	<0.001 (***)	0.057	<0.001 (***)
	Without HG sample (EF, F)	0.028	0.236	0.027	0.005 (**)

Significant results are in bold (***p < 0.001; **0.001 < p < 0.01; *0.01 < p < 0.05).
