# Supplementary material for: 11,000 years of craniofacial and mandibular variation in Lower Nubia
Source: Sci Rep. 2016 Aug 9;6:31040. doi: 10.1038/srep31040 (PMC4977491; doi:10.1038/srep31040)
Supplement: Supplementary Information [file srep31040-s1.doc]

**11,000 years of craniofacial and mandibular variation in Lower Nubia**

Manon GALLAND^1,2*^, Denis P. VAN GERVEN^3^, Noreen VON CRAMON-TAUBADEL^4^, Ron PINHASI^1^

^1^ School of Archaeology and Earth Institute, University College Dublin, Belfield, Dublin 4, Ireland

^2^ Muséum national d’Histoire naturelle – UMR 7206 - CNRS, 75016 Paris, France

^3^ University of Colorado, Boulder, United States

^4^ University at Buffalo, SUNY, Department of Anthropology, Buffalo, United States

**Supporting Information**

Table S1: Anatomical description of the cranial landmarks^1, 2, 3^

| **Landmark** | **Anatomical definition** | |
| --- | --- | --- |
| **Midline points** | | |
| Prosthion | Point on the maxillary bone where the midsagittal plane meets a tangent that goes through the alveolar margins of the central incisors. | |
| Nasospinale | Point where the midsagittal plane meets the inferior inner rim of the nasal aperture. | |
| Nasion | Midline point where the two nasal bones and the frontal intersect. | |
| Glabella | Intersection of the ridge curve on the arcus superciliaris with the midplane. | |
| Bregma | Midline point at the intersection of sutura sagittalis and sutura coronalis. | |
| Lambda | Point where the sagittal and lambdoid sutures meet. | |
| Inion | Midline point at the confluence of the lineae nuchae superiors. | |
| Opisthion | Midline point at the posterior margin of the foramen magnum. | |
| Basion | Midline point at the anterior margin of the foramen magnum. | |
| Sphenobasion | Point where the midsagittal plane intersects the sphenooccipital suture. | |
| Staphylion | Most posterior point on the interpalatal suture. | |
| Palate | Intersection of medial and lateral palatal sutures. | |
| Foramen incisivum | Point where the medial palatal suture meets the posterior margin of the foramen incisivum. | |
| **Bilateral points** | | |
| Asterion | | Point where the lambdoid, parietomastoid and occipitomastoid sutures meet. |
| Mastoideale | | Most inferior point on the mastoid process. |
| Auriculare | | Point vertically above the center of the external auditory meatus at the root of the zygomatic process. |
| Zygotemporale inferior | | Most inferior point on zygomaticotemporal suture. |
| Jugale | | Point in the depth of the notch between the temporal and frontal process of the zygomatic. |
| Frontomalare orbitale | | Point where the frontozygomatic suture crosses the inner orbital rim. |
| Frontomalare temporale | | Point where the frontozygomatic suture crosses the temporal line or the orbital rim. |
| Ectomolare | | Most lateral point on the outer surface of the alveolar margin of the maxillare |
| Zygomaxillare | | Most inferior point on the zygomaticomaxillary suture. |
| Zygoorbitale | | Point where the orbital rim intersects the zygomaticomaxillary suture. |
| Maxillofrontale | | Point where the anterior lacrimal crest of the maxilla meets the frontomaxillary suture. |
| Alare | | Most lateral point on the nasal aperture taken perpendicular to the nasal height. |
| Frontotemporale | | Point where the temporal line reaches its most anteromedial position on the frontal. |

Table S2: Anatomical description of the mandibular landmarks^1, 2, 3, 4^

| **Landmark** | **Anatomical definition** | |
| --- | --- | --- |
| **Midline points** | | |
| Infradentale | Most superior midline point on the buccal surface of the alveolus. | |
| Pogonion | Most anterior midline point on the mental eminence. | |
| Gnathion | Most inferior midline point on the mandibular symphysis. | |
| Mandibular orale | Most superior midline point on the lingual surface of the alveolus. | |
| Linguale | Most superiorposterior point on the linguale superior transverse torus. | |
| **Bilateral points** | | |
| Condyle tip | | Most anterior point on the superior surface of the mandibular condyle. |
| Condylion medial | | Most medial point on the superior surface of the mandibular condyle. |
| Condylion lateral | | Most lateral point on the superior surface of the mandibular condyle. |
| Mandibular foramen (superior) | | Most anterior, inferior point on the medial edge of the mandibular foramen. |
| Alveolus (posterior) | | Most superior, posterior point on the alveolus. |
| M3 (lateral posterior) | | Most lateral point on the alveolus posterior to M3. |
| M1M2 lateral | | Most lateral point on the alveolus posterior to the M1 and anterior to M2. |
| CP3 lateral | | Most lateral point on the alveolus between the canine and P3. |
| Mental foramen (anterior) | | Most anterior point on the lateral edge of the mental foramen. |
| Ramus (anterior and in line with alveolus) | | Most anterior point on the ascending ramus in line with the alveolus. |
| Gonion | | Point of maximum curvature on the posterior-inferior border where the posterior ramus and the corpus intersect. |
| Ramus (posterior and in line with alveolus) | | Most posterior point on the ascending ramus in line with the alveolus. |
| Sigmoid notch | | Most superior point of maximum inflection in the depth of the sigmoid notch. |
| Coronion | | Most superior point on the coronoid process. |

Table S3: Results of ANOVA tests performed on the centroid size for the cranium and the mandible. Significant results are in bold.

|  | **F** | **P** |
| --- | --- | --- |
| **Skull** | 2.49 | 0.0518 |
| **Mandible** | **7.749** | **1.99^e-5^ (***)** |

Table S4: Results of Tukey’s honestly significant differences (HSD) performed on the centroid size for the cranium (up) and the mandible (down). Significant results are in bold.

| **p adj.** | **A-group** | **C-group** | **Meroitic** | **Pharaonic** | **Mesolithic** |
| --- | --- | --- | --- | --- | --- |
| **A-group** |  | 0.9808410 | 0.9966873 | 0.9999454 | 0.1130186 |
| **C-group** | 0.9446303 |  | 0.8628507 | 0.9395995 | 0.1286542 |
| **Meroitic** | 0.9999945 | 0.9900016 |  | 0.9991343 | 0.0521838 |
| **Pharaonic** | 0.9883993 | 0.9991444 | 0.9984845 |  | 0.0631206 |
| **Mesolithic** | **0.0000479** | **0.0002677** | **0.0045745** | **0.0001992** |  |

Table S5: Results of linear regressions for the cranium with each principal components as dependent variable and the centroid size as independent variable. The 30 principal components represent 95% of the total variance.

| **CP** | **% variance** | **Adjusted R^2^** | **p** |
| --- | --- | --- | --- |
| 1 | 12.00 | **0.1337** | **0.0012** |
| 2 | 10.83 | -0.0134 | 0.7539 |
| 3 | 8.5 | -0.0013 | 0.3424 |
| 4 | 6.8 | -0.0147 | 0.8959 |
| 5 | 6.23 | -0.0145 | 0.87 |
| 6 | 5.6 | 0.0019 | 0.2922 |
| 7 | 5.44 | 0.0003 | 0.3163 |
| 8 | 4.44 | 0.0021 | 0.2887 |
| 9 | 4.1 | -0.0149 | 0.9748 |
| 10 | 4.0 | -0.0146 | 0.8903 |
| 11 | 3.14 | 0.0194 | 0.1301 |
| 12 | 2.85 | -0.0149 | 0.9464 |
| 13 | 2.58 | 0.0327 | 0.0737 |
| 14 | 2.1 | **0.0681** | **0.0172** |
| 15 | 1.85 | 0.0322 | 0.0752 |
| 16 | 1.67 | -0.0102 | 0.6055 |
| 17 | 1.63 | -0.0031 | 0.3778 |
| 18 | 1.47 | -0.0072 | 0.4757 |
| 19 | 1.43 | 0.0227 | 0.1128 |
| 20 | 1.25 | 0.0162 | 0.15 |
| 21 | 1.17 | 0.037 | 0.0616 |
| 22 | 1.0 | 0.0027 | 0.2797 |
| 23 | 0.93 | -0.0066 | 0.4589 |
| 24 | 0.86 | -0.0082 | 0.5067 |
| 25 | 0.77 | -0.0093 | 0.5437 |
| 26 | 0.71 | -0.0054 | 0.4296 |
| 27 | 0.60 | -0.0148 | 0.9389 |
| 28 | 0.53 | 0.0132 | 0.1713 |
| 29 | 0.52 | -0.003 | 0.3744 |
| 30 | 0.46 | -0.0137 | 0.7808 |

**Table S6:** Results of linear regressions for the mandible with each principal components as dependent variable and the centroid size as independent variable. The 24 principal components represent 95% of the total variance.

| **CP** | **% variance** | **Adjusted R^2^** | **p** |
| --- | --- | --- | --- |
| 1 | 20.78 | 0.0088 | 0.6851 |
| 2 | 12.71 | **0.0836** | **0.0024** |
| 3 | 11.28 | **0.0313** | **0.0455** |
| 4 | 8.5 | 0.0251 | 0.0654 |
| 5 | 7.24 | **0.0759** | **0.0037** |
| 6 | 4.7 | -0.002 | 0.3699 |
| 7 | 4.47 | -0.0097 | 0.7863 |
| 8 | 3.55 | **0.0672** | **0.006** |
| 9 | 2.87 | -0.0013 | 0.352 |
| 10 | 2.66 | -0.0084 | 0.6586 |
| 11 | 2.4 | -0.0059 | 0.5102 |
| 12 | 1.75 | -0.0104 | 0.9169 |
| 13 | 1.73 | 0.0004 | 0.3089 |
| 14 | 1.57 | -0.0088 | 0.6903 |
| 15 | 1.3 | -0.0091 | 0.7153 |
| 16 | 1.16 | 0.015 | 0.1195 |
| 17 | 1.06 | -0.0085 | 0.6635 |
| 18 | 1.01 | 0.0139 | 0.1286 |
| 19 | 0.92 | -0.0103 | 0.8926 |
| 20 | 0.79 | -0.0038 | 0.4274 |
| 21 | 0.76 | -0.0089 | 0.7015 |
| 22 | 0.68 | 0.01 | 0.1632 |
| 23 | 0.6 | 0.0095 | 0.1689 |
| 24 | 0.56 | -0.0051 | 0.4776 |

**References**

1. Bass, W.M. Human Osteology: A laboratory and field manual (Missouri Archaeological Society, Columbia, MO, 1995).

2. Bräuer, G. Osteometrie in *Anthropologie: Handbuch der vergleichenden Biologie des Menschen*, Band 1 (eds Knusmann, R. & Martin R.) 160-231 (Stuttgart, 1988).

3. White, T.D. & Folkens, P.A. The Human Bone Manual. (Elsevier, Amsterdam, 2005).

4. von Cramon-Taubadel, N. Global human mandibular variation reflects differences in agricultural and hunter-gatherer subsistence strategies. *Proc Nat Acad Sci* **108**, 19546-19551 (2011).
